# Evaluation of endovenous sedation using BIS monitoring in dentistry. A systematic review

**DOI:** 10.4317/medoral.22884

**Published:** 2020-06-10

**Authors:** Silvia Pérez-García, Naroa Lozano-Carrascal, Juan Antonio Ruiz-Roca, Pía López-Jornet, Jordi Gargallo-Albiol

**Affiliations:** 1 DDS, MS. International Master in Oral Surgery. Department of Oral and Maxillofacial Surgery. International University of Catalonia, Spain; 2 DDS, MS, PhD. International Master in Oral Surgery. Department of Oral and Maxillofacial Surgery. International University of Catalonia, Spain; 3 DDS, MS, PhD. Department of Special Patients. University of Murcia, Spain; 4 DDS, MS, PhD. Department of Oral Medicine. University of Murcia, Spain

## Abstract

**Background:**

The aim of the present review was evaluate the utility and validity of the Bispectral Index (BIS) in dental treatment carried out under endovenous sedation, and compare its efficacy with clinical sedation scales.

**Material and Methods:**

Electronic and manual literature searches were conducted by two independent reviewers for articles published up to April 2017 in several databases, including Medline and Cochrane Library.

**Results:**

Sixteen articles met the inclusion criteria. A correlation was identified between BIS and clinical sedation scales. A BIS range between 75 and 84 showed a high probability of corresponding to an Observer’s Assessment of Alertness and Sedation Scale (OAA/S) value of 3; a scored 3 on the Ramsay scale corresponds around 85 on the BIS; while BIS values between 57 and 64 corresponded to a University of Michigan Sedation Scale value of 3. BIS monitoring provides continuous measurement of the patient’s hypnotic state or state of consciousness, awareness, and recall. It proved impossible to perform an analysis of statistical data drawn from the studies reviewed due to the disparity of inclusion criteria among the works.

**Conclusions:**

BIS for sedation monitoring might make possible to evaluate sedation levels objectively in real time, reducing the dose of the sedative required, increasing safety, and minimizing secondary effects.

** Key words:**Bispectral analysis, BIS monitoring, intravenous sedation, dental treatment, anesthetics.

## Introduction

The term sedation describes a depressed level of consciousness, which varies from light (conscious sedation) to deep sedation accompanied by increasing depression of the physiological systems (1). Sedation is obtained using drugs of short to medium effect (1). As sedation deepens, there is an increasing likelihood of adverse events, and so the depth of sedation should be matched by adequate professional competence to ensure safety (2). Sedation reduces anxiety among patients facing surgical procedures (3), achieves a certain level of analgesia, prevents stress-related complications during dental treatment (4), and allows safe patient monitoring.

Oral drug administration is probably the simplest means of sedation, but this route is not sufficiently controllable to achieve the deeper levels of sedation required for working safely with extremely anxious patients, particularly children. Therefore, endovenous sedation is recommended in some situations, but this demands the additional support, expertise, and continuous supervision of a specialist (3). An anesthetist or trained specialist will observe the patient continuously, controlling the cardio-respiratory function using pulse oximetry to monitor arterial oxygen saturation and the heart rate (5,6). The depth of sedation may be monitored (7) by observing clinical signs (8,9) and applying some sedation criterion such as Verrill’s sign (partial drooping of the eye-lids) (10-12), and/or asking the patient if he/she feels relaxed or not (10,12), or by using different sedation assessment scales (10).

A wide variety of scales have been developed to assess the patient’s state of sedation continuously from consciousness to unconsciousness (13-15) in endovenous sedation. These scales aim to offer a standardized means of assessing the level of sedation in both research and clinical settings (7,9,13-15). The most popular sedation scale is the Observer’s Assessment of Alertness and Sedation Scale (OAA/S) (9), followed by the University of Michigan Sedation Scale (UMSS), and the Ramsay Sedation Scale (7).

The OAA/S measures the sedated subject’s alertness level based on four categories: responsiveness, speech, facial expression, and appearance of the eyes. The patient is scored for each category obtaining an overall score based on the highest level of alertness in each (9). But of course it is difficult to assess speech and facial expression when patients are undergoing dental treatment (9). The UMSS is an observational scale that assesses the level of alertness on a 5-point scale: 0 (awake) to 4 (unresponsive to deep stimulation) (7). The Ramsay scale assesses aspects that are identifiable visually: anxiety, agitation, whether eyes are open or closed, patient response to orders, visual or aural stimuli, with scores ranging from 1, when the patient is anxious, restless or both, to 6 when the patient is unresponsive to any stimuli (3).

Electroencephalograms (EEG) can also be used to assess the depth of sedation, providing an objective evaluation of the suppression of the central nervous system (CNS), but this is difficult to interpret clinically (16).

**Figure 1:**
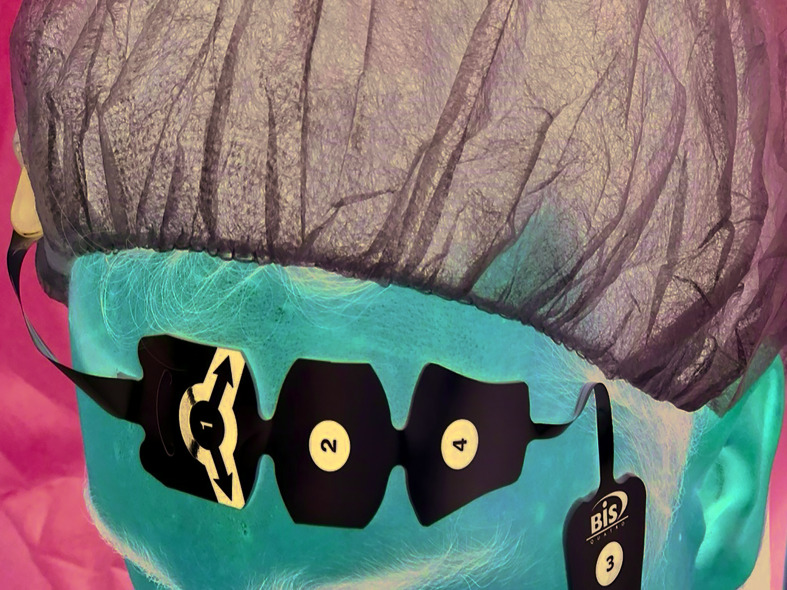
Clinical photograph of BIS electrodes placed on patient's forehead.

BIS is a neurophysiological monitoring parameter that has gained popularity in anesthetic practice in recent years (3) (Fig. 1). BIS (Aspect Medical Systems, Natick, Mass, USA) derives from bispectral analysis and monitors the effects of anesthesia based on electroencephalograms (EEG) (17). This was the first technology to be approved by the US Food and Drug Administration (in 1996) to aid in assessing the depth of anesthesia in adults (11,16). It makes a complex mathematical calculation of EEG data and is directly related to cortical activity (3), in which the shape of EEG waves changes with the patient’s level of alertness (5,11). The BIS is a dimensionless scale from 100 to 0, whereby 100 represents an awake clinical state, while 0 represents a total electric silence (complete cortical suppression) (16). General anesthesia comprises values range of 40-60, while deep sedation is within 60-70 and 70 to 90 represents light to moderate sedation (3). The patient is considered awake for values over 90 (11,18). A value between 65-70 and 80-85 has been recommended for conscious sedation (12,19) to reduce possibility of infra- or over-sedation, which runs risks of cardio-respiratory depression and increased recovery time (16).

The aim of this systematic review was to evaluate the use of BIS monitoring during endovenous sedation in patients undergoing dental treatment, comparing BIS with sedation scales.

## Material and Methods

- Patient, Intervention, Comparison, Outcome Studies (PICO(S)) Question

This systematic review fulfilled PRISMA criteria (Preferred Reporting Items for Systematic Reviews and Metanalyses), and PICO(S) questions were applied as assessment criteria to identify the Patient or Population, Intervention, Control and Comparison, Outcome, and Study types (20,21):

P: patients undergoing dental treatment 

I: dental treatment performed under endovenous sedation monitored by BIS

C: evaluation of the patient’s sedation level using BIS monitoring in comparison with subjective assessment scales.

O: the primary results were the BIS values registered during dental treatment under endovenous sedation; secondary results were the relationship between BIS values and the values obtained in subjective sedation assessment scales.

S: prospective or retrospective clinical studies.

- Eligibility Criteria

Articles were included in this systematic review if they met the following criteria: 1) clinical studies in humans; 2) sample of at least 10 patients; 3) patients older than 3 years and younger than 65 years; 4) randomized and non-randomized prospective studies, cohort studies and retrospective studies; 5) studies of oral/dental treatments performed under endovenous sedation. Consequently, the exclusion criteria consisted of: 1) studies written in languages other than English; 2) review articles, letters, editorials, doctoral theses or abstracts; 3) studies involving treatments performed under general anesthesia and or inhalation sedation; 4) studies in which the intervention performed was not oral.

- Information sources and search strategy

Electronic and manual literature searches, conducted by two independent reviewers (S.P. and N.L.), covered studies until April 2017 across the National Library of Medicine (MEDLINE by Pubmed and the Cochrane Library using different combinations (and Boolean Operators: AND and OR) of the following search terms/MeSH/key words: “bispectral monitoring” [MeSH term] OR “bispectral analysis” [MeSH term] OR “bispectral index” [MeSH term] AND “dental” [MeSH term] OR “dental treatment” [MeSH term] OR “oral surgery” [MeSH term] OR “implants” [MeSH term].

The screening process consisted of three steps: firstly, by title; secondly, by reading the abstract; and thirdly, by reading the full text. The information extracted from each of the articles analyzed was entered in a Microsoft Excel Office® spreadsheet (Microsoft Corporation Redmond, USA)

Studies were excluded independently by screening the titles and abstracts by two investigators (S.P. and N.L.), and the final eligibility of an article was confirmed after discussion. In case of disagreement, and additional investigator (J.G.) was consulted with for reaching and agreement. The definitive stage of screening involved full-text reading using the predetermined data extraction form to confirm the eligibility of each study based on the previously mentioned inclusion and exclusion criteria.

- Data extraction

The information extracted from each article included: 1) author, year of publication and study type; 2) methods (comparison); 3) Dental treatment; 4) Patient sample characteristics (number of patients, women: men, mean years age, range years age, ASA category); 5) drugs used for sedation; 6) variables registered; 7) sedation assessment scales used; 7) complications; 8) study conclusions.

## Results

- Study selection

**Figure 2:**
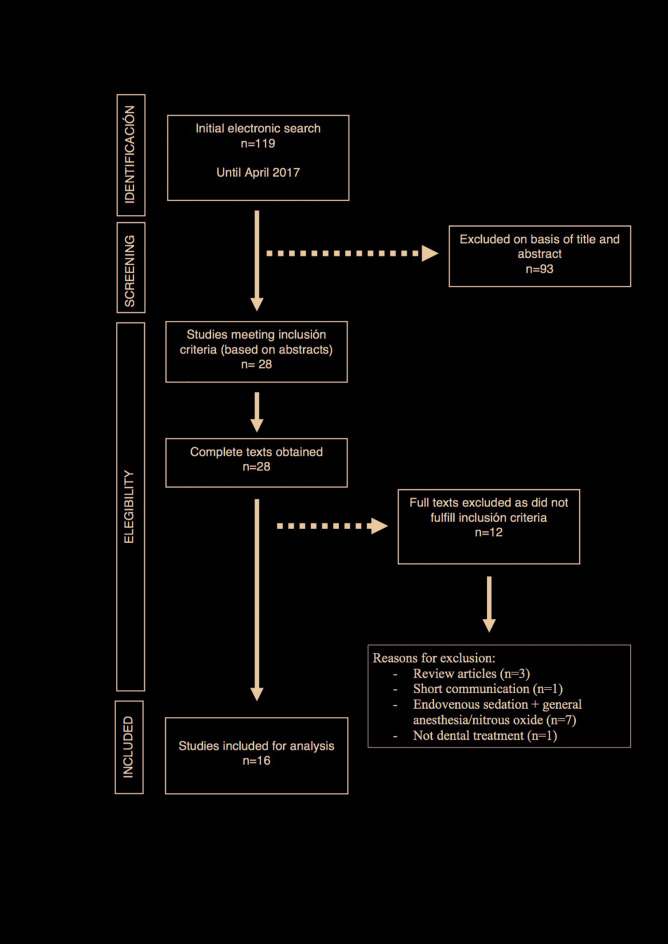
PRISMA flow diagram of selection process.

The initial database search identified a total of 119 articles of which 28 were considered to fulfill the inclusion criteria after assessing the titles and abstracts (with an agreement level between reviewers of 86.41%; kappa=0.63) and so the full text was read in depth. Twelve articles were excluded after reading the full text, as they did not fulfill the inclusion criteria. The reasons for excluded articles were: review articles (22-24), one short communication (25), no dental treatment performed (26) and treatments performed under general anesthesia or nitrous oxide and/or endovenous sedation (7,27,30). Manual searches and cross-referencing did not identify any further works and so the final selection included a total of 16 articles (3-6,9-11,13,16,18,19,31-36) (Fig. 2).

- Characteristics of the works analyzed

All characteristics of the articles reviewed are shown in Table 1. Of these, two studies involved non-cooperative children aged under 8 years (4,18). The rest of the studies involved adult patients (3,5,6,9,10,12,16,19,31-36). All patients were ASA category I-II (American Society of Anesthesiologists) (37), with the exception of one investigation that did not provide this information (33,34). Studies by Ishii *et al*. (34) and Sakaguchi *et al*. (31) conducted studies on adults with intellectual disability.

**Table 1:**
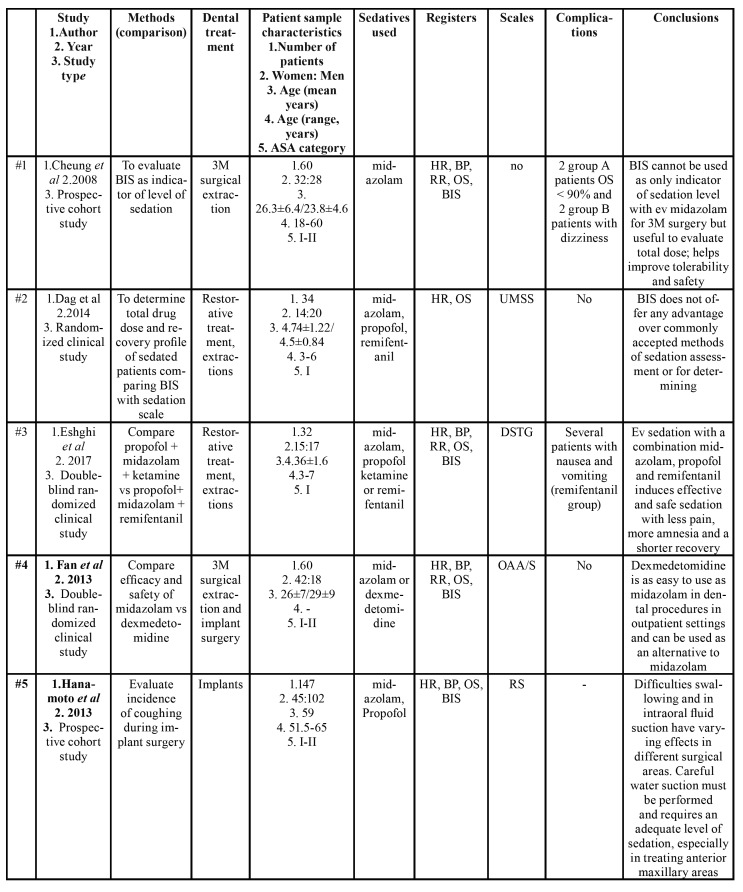
Characteristics of articles included for qualitative synthesis.

**Table 1 cont.**
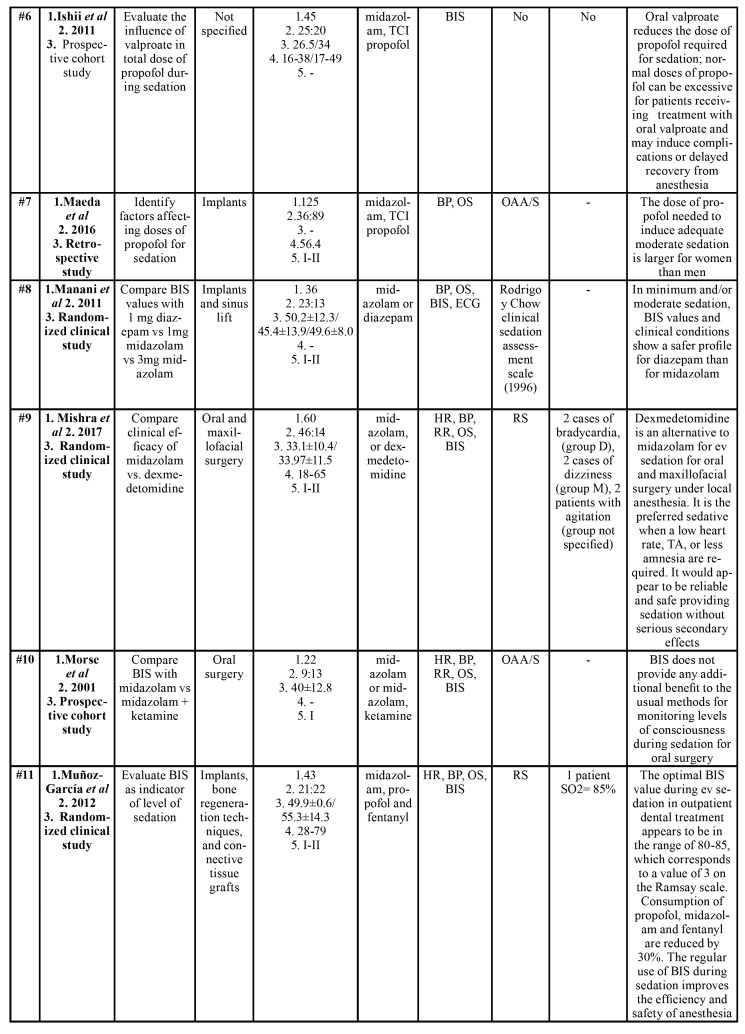
Characteristics of articles included for qualitative synthesis.

**Table 1 cont.**
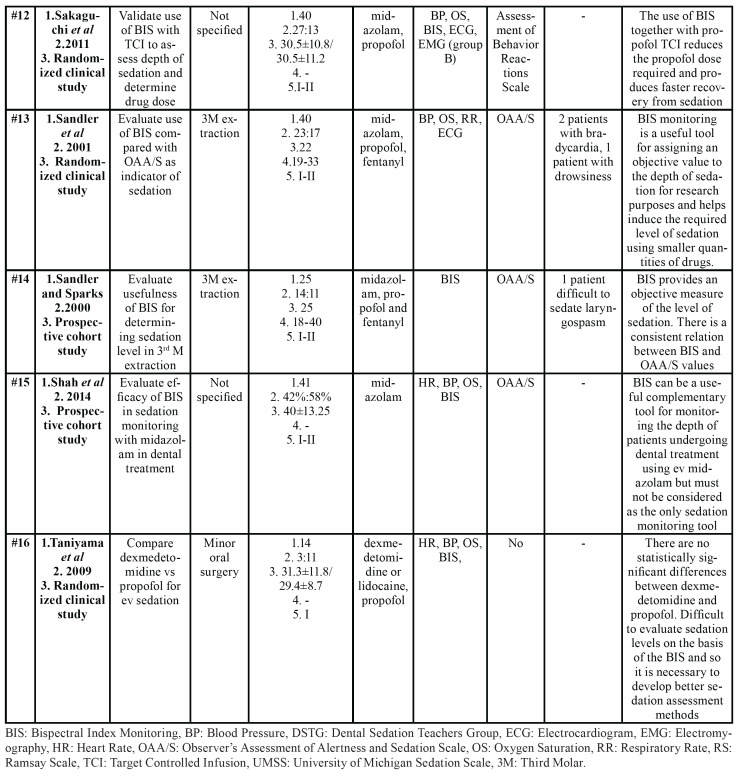
Characteristics of articles included for qualitative synthesis.

In seven studies, the objective was to assess the validity of BIS monitoring in endovenous sedation in patients undergoing dental treatment (3-5,9,12,19,31); in the other seven, sedation monitoring was used to compare different sedative drugs (10,16,18,32,34-36). In the studies by Maeda *et al*. (6), Hanamoto *et al*. (33) and Ishii *et al*. (34), BIS was used as one more method of patient monitoring along with blood pressure, heart rate, etc.

Fifteen works were prospective studies (3-5,9,10,12,16,18,19,31-36) and one was retrospective (6). Of the prospective studies, nine were randomized clinical studies (3,4,10,18,19,31,32,35,36), and six were prospective cohort studies (9,12,16,18,33,34). In addition, all were conducted at a single center, most of them in Asia, and more than half in Japan.

Oral/maxillofacial or implant surgeries were the most frequent procedures (3,9,10,12,16,19,32,33,35,36), followed by conservative dental treatments or extractions (4,18). Three works did not stipulate the type of dental treatment performed (5,31,34).

- Comparisons between sedation scale values and BIS values 

A strong positive relation was observed between BIS values and other sedation scale scores (OAA/S, the Ramsay scale, and the UMSS) in four studies (3-5,9).

The most widely used scale in the studies reviewed was the Observer’s Assessment of Alertness/Sedation (OAA/S) (5,9,16,19,32), followed by the Ramsay sedation scale (3,33,35). Other scales used included the University of Michigan Sedation Scale (UMSS) (4), the “Assessment of Behavior Reactions Scale” (ABR) (31), Clinical assessment of sedation (10) and the “Dental Sedation Teachers Group” (DSTG) (18).

The correlation between BIS value and sedation scales are described in various studies: the BIS value that corresponded to an awake state in the OAA/S (5 points) was 95-99, medium sedation or relaxation (4 points) corresponded to 75-84, and deep sedation (3 points) corresponded to 70-79 (5); for Shah *et al*. (5) a BIS range between 75 and 84, showed a high probability of corresponding to an OAA/S value of 3; For Sandler and Sparks (9) differentiation between levels of sedation was clear, except for making a distinction between 2 and 3 on the OAA/S; the Ramsay sedation scale and BIS assessment stabilized 5 minutes after commencing sedation and scored 3 on the Ramsay scale and around 85 on the BIS, remaining sTable until the intervention had been completed (3); finally, Dag *et al*. also found a clear correlation between mean BIS values and the UMSS, whereby BIS values between 57 and 64 corresponded to a UMSS value of 3 (4).

- Use of the BIS for comparing the different sedatives used in dentistry

Some investigations used the BIS as an objective instrument for measuring sedation and did not doubt its efficacy or the accuracy of readings, and so were confident in using the BIS to compare the efficacy of different drugs for endovenous sedation (10,18,32,35). In this way, they can determine which drug is the safest and most effective in groups of patients undergoing a specific treatment (10,18,32,35). The BIS scores descend gradually after drug administration and then remains between 80 and 85, the optimal level of sedation (35,36).

Contrary Bispectral analysis during deep sedation of pediatric oral surgery patients did not bring any benefit in comparison with the established methods of conscious sedation assessment for both Taniyama *et al*. (36) and Morse *et al*. (16). Morse *et al*. found the BIS a unuseful method because mean BIS values were 90 for their midazolam group and 94 for the midazolam-ketamine group and these did not vary much over time from the patients’ baseline level, except immediately after inducing sedation when values dropped to 85 (16). This would mean that the patient reaches a state of temporary deep sedation but that this would not be produced if the drug was administered by means of continuous slow infusion (16).

- Results of BIS monitoring

Two articles reported numerical data obtained from BIS monitoring (18,35), used to determine which minimum and maximum values are adequate for patients undergoing dental treatment (Table 2). These were maintained at 63.01 (5 minutes after beginning treatment) and 78.65 (maximum value obtained 45 minutes after beginning treatment) obtaining an overall mean of 70.64. The minimum BIS value [38.05] was obtained in the ketamine group and the maximum BIS value [92.48] in the dexmedetomidine group, 45 minutes after the start of the procedure.

**Table 2:**
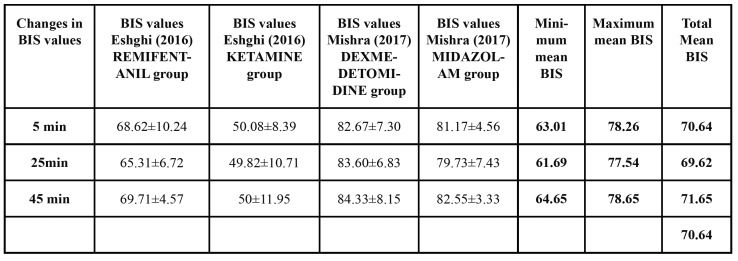
BIS numerical data.

## Discussion

Most of the methods used to estimate the depth of anesthesia are based on subjective scales that assess patient responses, often through stimulation of the patient by means of verbal or physical contact (23).

The evaluating scales suffer a major limitation in that they are based on a clinician’s subjective judgment (7,11,12,19,38). This is particularly difficult in the context of intraoral procedures, as the patient is unable to respond to verbal stimuli (9,12,38). The introduction of new sedative agents and the use of multiple drugs to induce a state of anesthesia mean that the most reliable instrument for providing objective information about the level of anesthesia during conscious sedation is the electroencephalogram (EEG) or the bispectral index (BIS) (8).

BIS value is inversely correlated to the depth of sedation so that a drop in BIS value represents a deeper level of sedation (24). In this way, it differentiates between deep sedation and lighter levels but runs into difficulty distinguishing between moderate and deep sedation (9,24,39), a distinction that requires a certain level of clinical experience (24,39).

In the present review, two works provided numerical data registered by the BIS (18,35) providing a quantitative measure of the levels of sedation induced, without the need to stimulate the patient (35). In agreement with Cheung *et al*. (12), who recommend a BIS value of between 65 and 85, patients in these two studies (18,35) remained in a state of moderate or deep sedation presenting mean BIS values of 70.64 (18,35). The minimum BIS value registered was 38.05 (18) (ketamine group 45 minutes into the intervention), which fell within the BIS range corresponding to a deep hypnotic state close to general anesthesia (5), while the maximum value of 92.48 (35) registered (dexmedetomidine group 45 minutes into the intervention) represents a state of minimum sedation or anxiolysis (10).

Taniyama *et al*. (36) found that BIS gradually dropped to 80-85 at the moment of optimal sedation, a similar observation to Mishra (35), who showed that the drugs tested had an optimal sedative effect and induced adequate sedation levels. But these results contradicted a work by Morse *et al*. (16) which found that the BIS value did not alter significantly from the baseline level to the end of the dental procedure, remaining at around 90, a finding that places the efficacy and usefulness of BIS monitoring in some doubt.

The present review observed a drop in mean BIS value at 25 minutes into the procedure to 69.62 (deep sedation), regardless of the sedative drug employed, from which the patient began to recover after 45 minutes, close to the end of the treatment. In nine articles, the authors represented the BIS values registered as graphs, making it impossible to extract precise values for analysis (3-5,9,11,12,16,19,32,36). Five articles described BIS monitoring but without supplying numerical data or even expressing these as graphs (6,10,31,33,34,40). This imposed a limitation on the present review in terms of data analysis that might point to firm conclusions.

Some investigations (1,3,12) related the use of the BIS with a general reduction in the incidence of complications. Muñoz-Garcia *et al*. (3) found that the use of BIS monitoring led to 30% reduction in endovenous sedative consumption, reducing the probability of secondary effects, and reducing the economic cost of procedures (3), an observation that concurs with the study by Sandler *et al*. (19). Although not all the studies reviewed mention complications associated with sedation/anesthesia (5,10,11,16,31,33,35,36), the most serious complications during oral treatment are associated with respiratory depression and hypoxemia (12), followed by nausea and vomiting (3). Bradycardia or persistent post-operative drowsiness can also be important complications (19). The incidence of complications in the studies under review was 1.82%. The most common was dizziness (26.66%) (12,35) and bradycardia (26.66%) (19,35), SaO2 < 90% (20%) (3,12), agitation (13.33%) (35) and lastly, drowsiness (6.66%) (19) and laryngospasm (6.66%) (9). Eshghi *et al*. (18) reported nausea and vomiting but did not stipulate the number of cases presenting these complications.

This review showed that with the use of the BIS for sedation monitoring, it is possible to evaluate sedation levels objectively (9,24,39) in real time (19,24,37,39), eliminating the need for clinical evaluation (24,39). This is very important in the field of dentistry, as the presence of intraoral instruments makes it difficult to communicate with the patient in order to assess the level of sedation (24,39).

Despite the advantages of BIS monitoring mentioned by some authors, for others its use remains controversial. One of its disadvantages in the field of dentistry is that the device’s sensor is place on the forehead, close to the working area, which means that it is easy to provoke some interference in muscular activity or distortion of BIS readings as a result of high-frequency electric apparatus (3,5) although the most recent generation of BIS monitors have been designed to eliminate the majority of artifacts, but further research is needed to obtain definitive data(3). Some authors believed that BIS monitoring does not offer any advantage over the traditional methods used for sedation assessment and felt that it could not be relied on as the sole means of indicating the level of endovenous sedation (4,12,16). Another factor to bear in mind is the cost per patient of the BIS electrode, which varies from manufacturer to manufacturer between 15 and 40 USD (24,39).

The present systematic review presents some limitations. Although a comprehensive search strategy was employed, analysis of statistical data drawn from the studies reviewed proved impossible due to the disparity of inclusion criteria among the works, which derived from the different objectives. The sedative drugs used differed from study to study. Most used an established sedation regime involving various sedatives, the most frequent being a combination of midazolam and propofol (6,31,33,34), with the addition, in some cases, of fentanyl/remifentanil (3,4,9,18,19). Four of the works set out to compare two sedatives used during dental procedures, and so only used a single drug as inducer and maintainer (10,16,18,32,35,36). All the studies used midazolam alone or in combination with other sedatives except Taniyama *et al*. who did not use midazolam in any study group (36). Additionally, the studies also differ in the dental treatment performed, the sample sizes, and the patient age groups (adults and children).

In conclusion, BIS monitoring of conscious sedation offers better safety, particularly when endovenous sedation techniques are applied in a non-hospital operating theatre setting. Using BIS monitoring as an everyday working tool to manage patients’ level of consciousness might increase the efficiency of anesthesia, and probably reduce the incidence of complications. Nevertheless, further research within the field of dentistry is needed to confirm these advantages and to overcome the limitations identified in the works analyzed in this review.
